# A new class of chiral materials hosting magnetic skyrmions beyond room temperature

**DOI:** 10.1038/ncomms8638

**Published:** 2015-07-02

**Authors:** Y. Tokunaga, X. Z. Yu, J. S. White, H. M. Rønnow, D. Morikawa, Y. Taguchi, Y. Tokura

**Affiliations:** 1 RIKEN Center for Emergent Matter Science (CEMS), Wako, 351-0198, Japan; 2 Laboratory for Neutron Scattering and Imaging, Paul Scherrer Institute, CH-5232 Villigen, Switzerland; 3 Laboratory for Quantum Magnetism (LQM), École Polytechnique Fédérale de Lausanne (EPFL), CH-1015 Lausanne, Switzerland; 4 Department of Applied Physics, University of Tokyo, Bunkyo-ku 113-8656, Japan

## Abstract

Skyrmions, topologically protected vortex-like nanometric spin textures in magnets, have been attracting increasing attention for emergent electromagnetic responses and possible technological applications for spintronics. In particular, metallic magnets with chiral and cubic/tetragonal crystal structure may have high potential to host skyrmions that can be driven by low electrical current excitation. However, experimental observations of skyrmions have been limited to below room temperature for the metallic chiral magnets, specifically for the MnSi-type *B*20 compounds. Towards technological applications, transcending this limitation is crucial. Here we demonstrate the formation of skyrmions with unique spin helicity both at and above room temperature in a family of cubic chiral magnets: *β*-Mn-type Co-Zn-Mn alloys with a different chiral space group from that of *B*20 compounds. Lorentz transmission electron microscopy, magnetization and small-angle neutron scattering measurements unambiguously reveal formation of a skyrmion crystal under application of a magnetic field in both thin-plate and bulk forms.

## Abstract (web summary)

Magnetic skyrmions are circulating topologically-protected spin textures which can arise in chiral magnets and potentially be applied in information processing. Here, the authors demonstrate the appearance of skyrmions at and above room temperature in a *β*-Mn-type Co-Zn-Mn alloy.

## Graphical abstract

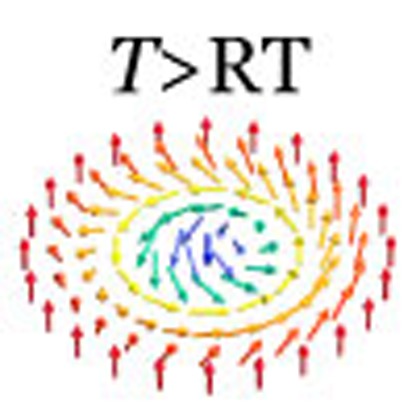

Skyrmions with topologically non-trivial spin textures^1^,^2^,^3^ have been observed or proposed to exist in various magnets due to different mechanisms, such as magnetic dipolar interaction^4^,^5^, antisymmetric spin exchange or so-called Dzyaloshinskii–Moriya (DM) interaction^6^,^7^,^8^,^9^, frustrated exchange interaction^10^ and four-spin exchange interactions^11^. In metallic compounds, skyrmions induce fictitious magnetic fluxes acting on the conduction electrons, thereby inducing the so-called topological Hall effect^12^,^13^,^14^. Conversely, an applied electric current can induce a flow of skyrmions, with a threshold current density (∼10^6^ A m^−2^), which is 5–6 orders of magnitude smaller than that necessary for driving magnetic domain walls in ferromagnets^15^,^16^,^17^. This property can be understood to arise from the theoretically predicted topological features of skyrmions^18^,^19^,^20^. On the other hand, in the insulating chiral magnet Cu_2_OSeO_3_, each skyrmion carries an electric dipole or quadrupole, which makes it possible to manipulate skyrmions by means of electric fields^21^,^22^,^23^,^24^,^25^. These features are promising for technological applications, including spintronic devices and ultra-low power-consumption, high-density magnetic memory.

Among the various skyrmions, those induced by the DM interaction are particularly interesting due to their relatively small size (<150 nm) and fixed unique helicity (spin swirling direction). Theoretically, a number of helical magnets mediated by the DM interaction and with non-centrosymmetric crystallographic symmetries are predicted to be good candidates for hosting stable skyrmions^2^,^26^. In spite of such anticipation, experimental observations of skyrmions in non-centrosymmetric bulk magnets have been limited to only *B*20-type alloys^3^,^6^,^7^,^27^ and Cu_2_OSeO_3_ (refs 21, 22), all of which have the same cubic chiral space group *P*2_1_3. Their helical transition temperatures (*T*_c_), and hence the maximum formation temperatures of skyrmions, are below room temperature (at most ∼278 K in FeGe^27^). The sub-ambient *T*_c_ is a bottleneck for the integration of skyrmions in non-centrosymmetric magnets into practical spintronics devices. Therefore, new systems with higher *T*_c_ are required.

One promising strategy to discover new DM helimagnets that host skyrmions above room temperature is to revisit non-centrosymmetric crystals that have been reported to be ferromagnetic above room temperatures. Similar to the case of *B*20 alloys^6^,^7^,^27^ and Cu_2_OSeO_3_ (refs 21, 22), the DM interaction combined with the chiral crystal structure might turn an otherwise ferromagnetic phase into a helimagnetic one with long magnetic periodicity, and thus possibly host magnetic skyrmions under the magnetic field.

One such example is Co_10_Zn_10_ with *β*-Mn-type structure^28^,^29^, which belongs to another cubic chiral space group *P*4_1_32 or *P*4_3_32, depending on its handedness, and which contains 20 atoms in the unit cell distributed across two kinds of crystallographic sites. One is 8*c* with the three-fold site symmetry, and which is occupied by Co atoms, while the other is 12*d* with the two-fold site symmetry and occupied by both Zn and Co atoms^29^. Co_10_Zn_10_ has been reported to be a ferromagnet with *T*_c_ ∼420 K and the Co magnetic moments (∼0.85 *μ*_B_ per Co atom) ordered along <100> according to neutron diffraction measurements^29^. It has also been reported that the Co-Zn-Mn ternary system crystalizes in the *β*-Mn-type structure over a relatively wide composition range, and with *T*_c_ dependent on the compositional ratio^30^.

In this study, a series of *β*-Mn-type Co-Zn-Mn alloys are prepared and investigated in detail. As a result, the formation of chiral skyrmions is unambiguously demonstrated in both bulk and thin plate forms at and above room temperature. These results overcome limiting factors in terms of both operating temperature and materials variation, and hence can be an important step, towards the technological application of magnetic skyrmions for spintronics devices.

## Results

### Temperature dependence of magnetization

**Figure 1:**
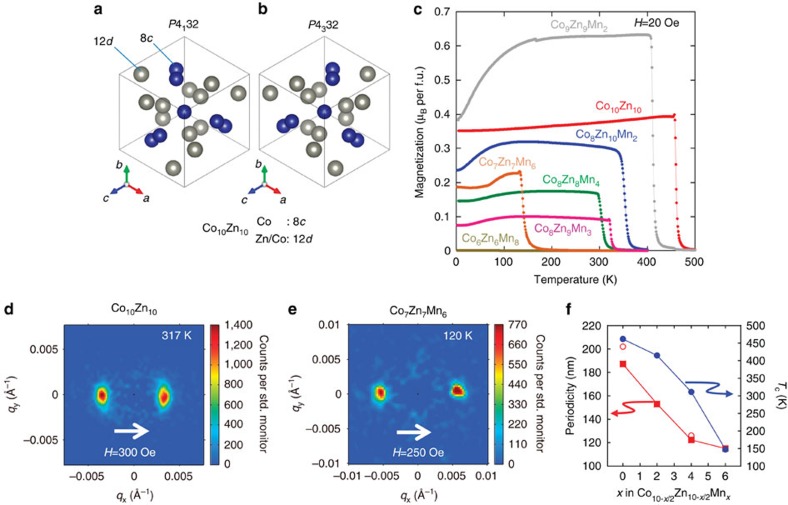
Crystal structure and helical magnetic order in the *β*-Mn-type Co-Zn-Mn alloys. (**a**,**b**) Schematic of *β*-Mn-type structure with (**a**) *P*4_1_32 and (**b**) *P*4_3_32 space group, dependent on its handedness (chirality). It contains two crystallographic sites, that is, 8*c* with the three-fold site symmetry occupied by Co atoms (indicated by blue circle) and 12*d* with the two-fold site symmetry occupied by both Zn and Co atoms (indicated by grey circles) in the case of Co_10_Zn_10_ (ref. 29). (**c**) Temperature dependence of the magnetization for a series of polycrystalline Co-Zn-Mn alloys measured at *H*=20 Oe on cooling. (**d**,**e**) Small-angle neutron scattering (SANS) images for polycrystalline samples of Co_10_Zn_10_ and Co_7_Zn_7_Mn_6_ at *T*=317 K, *H*=300 Oe and *T*=120 K, *H*=250 Oe, respectively. The magnetic field direction is perpendicular to the neutron beam, which is itself into the page. (**f**) Composition dependence of the helical ordering temperature (filled blue circles) and helical pitch obtained by the SANS measurements (filled red squares) and LTEM observations (open red circles), respectively.

All prepared samples were confirmed to be of *β*-Mn-type, whose structure is depicted in Fig. 1a,b. Figure 1c shows the temperature dependence of the magnetization for selected samples measured in field-cooling runs. *T*_c_ for Co_10_Zn_10_ is ∼462 K, which is higher than the reported value (∼420 K, ref. 29), possibly due to a slight difference in the composition. On substituting Co and Zn with Mn, *T*_c_ systematically decreases and Co_6_Mn_6_Zn_8_ no longer shows ferromagnetic-like behaviour. Magnetization measured at this magnetic field (*H*=20 Oe) is observed to decrease at low temperatures in many samples, but this tendency is mostly suppressed for *H*=1 kOe. Whether there is a second transition or not at low temperature remain a topic for future investigations.

### Demonstration of the helimagnetic nature

To elucidate the possible helical nature of the magnetic ordering in these compounds, we have performed small-angle neutron scattering (SANS) measurements. Figure 1d,e show typical SANS images obtained from polycrystalline pieces of Co_10_Zn_10_ and Co_7_Zn_7_Mn_6_ under respective magnetic fields of 300 and 250 Oe applied perpendicular to the incident neutron beam direction. In the vicinity of the *Q*=0 position, pairs of magnetic reflections are clearly observed nearly along the magnetic field direction, demonstrating the helical (or more precisely, conical due to the presence of the magnetic field) nature of the spin ordering in this system. The slight tilting of the peaks relative to the horizontal field direction demonstrates presence of weak magnetic anisotropy. As shown in Fig. 1f, the helical periodicity (*λ*) determined from the positions of the magnetic reflections shows a systematic change from *λ*∼185 nm for Co_10_Zn_10_ to *λ*∼115 nm for Co_7_Zn_7_Mn_6_. This can be consistently understood as a decrease in the dominant ferromagnetic exchange interaction (*J*), which is reflected in the decrease of *T*_c_. As a result, the periodicity which is given by *J*/*D* (where *D* is the DM interaction) also decreases. It is to be noted that all the compounds of Co_10-*x*/2_Zn_10-*x*/2_Mn_*x*_ are good metals showing resistivity of 140–280 μΩcm at room temperature. A detailed transport study is currently in progress.

### Formation of the chiral skyrmion above room temperature

Since the system is found to be a cubic DM helimagnet, the formation of skyrmions is anticipated on application of the magnetic field in analogy to the case of *B*20-type (MnSi-type) alloys^6^,^7^,^27^ and Cu_2_OSeO_3_ (refs 21, 22). To demonstrate directly the formation of skyrmions in this system, real-space observations by Lorentz transmission electron microscopy (LTEM) have been performed for a thin-plate sample with thickness of <150 nm. In LTEM the under- and over-focusing of the deflected electron beam can reveal magnetic contrast, which has proven an efficient way to investigate skyrmion textures^7^. A thin-plate specimen was sliced out from a polycrystalline ingot consisting of single-crystalline grains as large as several tens to hundred μm^3^, and LTEM observations were performed for single-crystalline areas of larger than 100 μm^2^ with specific crystallographic axes fortuitously normal to the thin plate.

**Figure 2:**
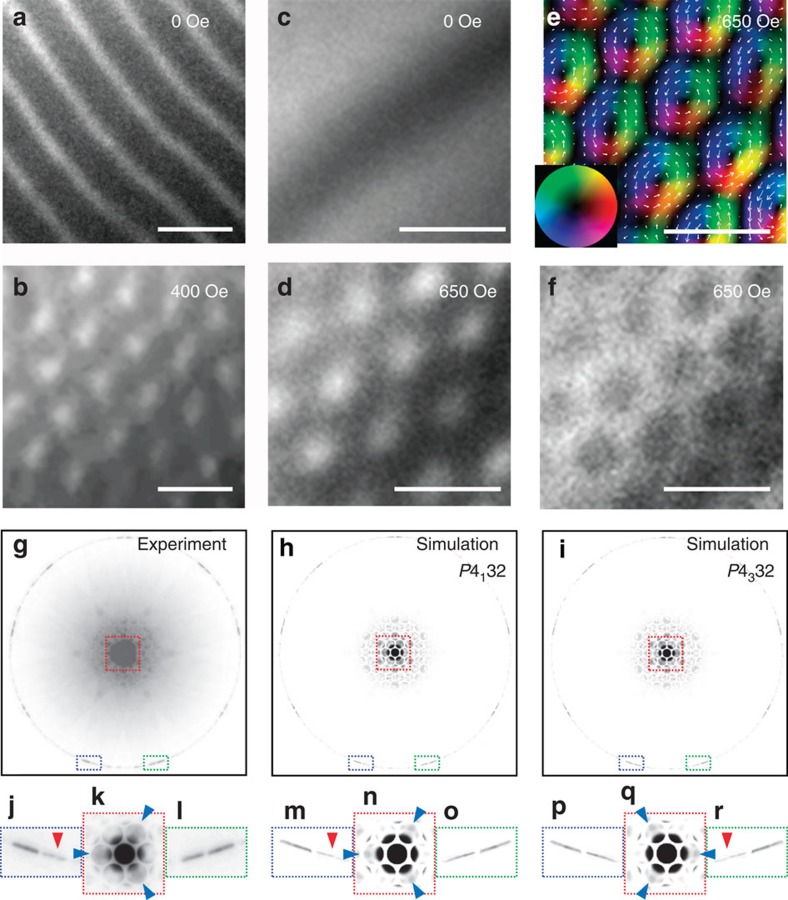
Lorentz transmission electron microscopy (LTEM) of skyrmion crystal. (**a**,**b**) LTEM image at 283 K on (111) plane of Co_8_Zn_8_Mn_4_ taken with under-focused condition at *H*=0 and 400 Oe, respectively. (**c**–**f**) LTEM image at 345 K on (110) plane of Co_8_Zn_10_Mn_2_ taken with under-focused condition obtained at (**c**) *H*=0 and (**d**) 650 Oe, and (**f**) with over-focused condition at *H*=650 Oe. In **c**, the dark band running diagonally across the image is the diffraction contrast and not of magnetic origin. (**e**) Colour map of in-plane components of the magnetic induction (which reflects in-plane magnetization) at *H*=650 Oe deduced from TIE analysis of the images (**d**) and (**f**). (**a**–**f**) Scale bars, 200 nm. (**g**) Observed convergent-beam electron diffraction (CBED) pattern of Co_8_Zn_10_Mn_2_ taken with the [111] incidence from the same specimen area where the LTEM images (**c**–**f**) were obtained. (**h**,**i**) Simulated CBED patterns assuming the space group No. 213 (*P*4_1_32) and No. 212 (*P*4_3_32), respectively. (**j**–**l**), (**m**–**o**) and (**p**–**r**) are magnified images for the selected areas (surrounded by blue, red and green dotted rectangles) of **g**,**h** and **i**, respectively. (**k**) is based on another picture with different exposure time for the purpose of avoiding intensity saturation. Characteristic structures to be compared between experimental result and simulations are indicated by blue and red triangles in **j**–**r**. Magnified images of the simulated pattern show significant differences for each crystal chirality. The simulated CBED pattern using *P*4_1_32 (see **m**–**o**) shows a good agreement with the experimental one (see **j**–**l**).

Figure 2a,b show the LTEM images for the (111) grain of Co_8_Zn_8_Mn_4_ taken in the under-focus condition at 283 K. At *H*=0 (Fig. 2a), a magnetic stripe structure is observed with a periodicity (*λ*∼124 nm) that is in excellent agreement with the SANS result (*λ*∼125 nm). On applying *H*=400 Oe normal to the plate, the emergence of a skyrmion crystal (SkX) is clearly observed (Fig. 2b).

Figure 2c–f show the LTEM images obtained for a (110) grain of Co_8_Zn_10_Mn_2_ at 345 K. Different from the former case of Co_8_Zn_8_Mn_4_, there is no discernible magnetic contrast at *H*=0 in the region shown in Fig. 2c in spite of *T*<*T*_c_, and magnetic stripes develop only in limited areas at this temperature (not shown). Considering the fact that LTEM is only susceptible to the in-plane components of the magnetic induction, which reflects in-plane magnetization that does not cancel out when summed up along the thickness direction of the plate, a possible assignment of this phase is a helix with propagation vector directed normal to the plane, as conjectured from observations in the bulk *B*20 compounds^6^,^31^. On application of magnetic field, however, a SkX emerges despite the absence of a helical structure with in-plane propagation vector at *H*=0. Figure 2d,f are under- and over-focused images, respectively, taken at *H*=650 Oe, where the contrast is inverted from each other, confirming their magnetic origin. Figure 2e is a colour map of the in-plane magnetic induction (reflecting magnetization) components deduced from Fig. 2d,f by use of a transport-of-intensity equation analysis^32^. Notably, the formation temperature (e.g., 345 K) of skyrmions for Co_8_Zn_10_Mn_2_ here is well above room temperature.

Convergent-beam electron diffraction (CBED) patterns can be utilized to assign the crystal chirality of the region where the LTEM image of Co_8_Zn_10_Mn_2_ was taken. Figure 2g shows the experimental CBED pattern with the [111] incidence, which has three-fold symmetry. The simulated CBED patterns for the possible crystal chiralities of *P*4_1_32 and *P*4_3_32 are shown in Fig. 2h,i, respectively. The magnified images (Fig. 2j–r) display significant differences that are dependent on the crystal chirality. The CBED pattern obtained experimentally (Fig. 2g) shows good agreement with the simulated pattern for the crystal chirality of *P*4_1_32 (Fig. 2h). The observed region was further shown to be a single crystallographic domain, and therefore of unique crystal chirality. This unique crystal chirality is hence reflected by the unique sign of the DM interaction so that the magnetic helicity of the skyrmions is fully aligned over the whole region (Fig. 2e), similiar to the case of the other cubic chiral magnets^7^,^21^,^27^.

### Magnetic phase diagrams in the bulk and thin-plate form

**Figure 3:**
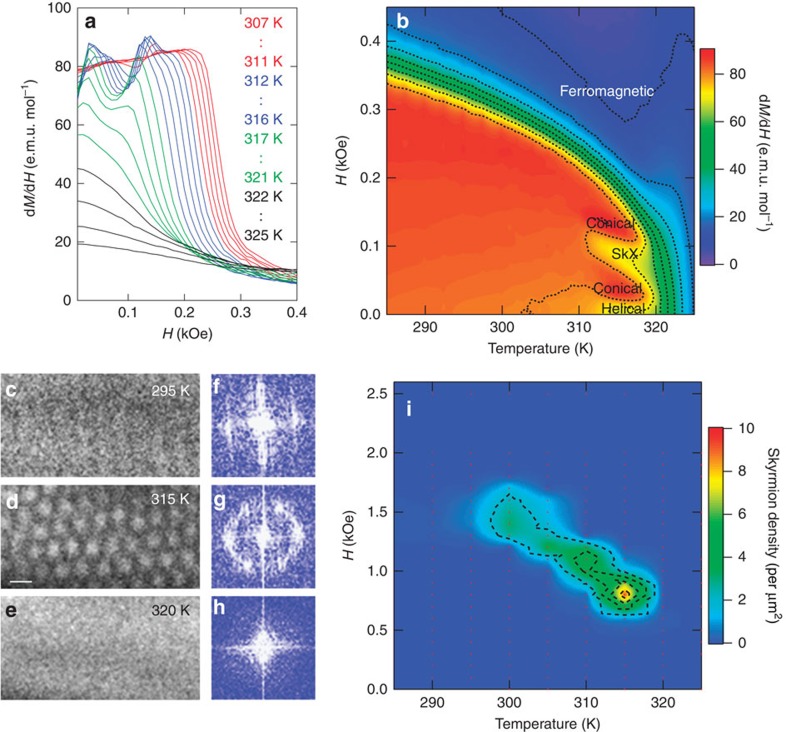
Magnetic phase diagrams of bulk and thin-plate forms. (**a**) Temperature dependence of isothermal d*M*/d*H* versus *H* curves for a polycrystalline piece of Co_8_Zn_9_Mn_3_. (**b**) Magnetic phase diagram of the bulk sample in magnetic field versus temperature plane as deduced from d*M*/d*H* curves. (**c**–**e**) LTEM images on (111) plane of Co_8_Zn_9_Mn_3_ taken with under-focused condition in *H*=0.7 kOe applied normal to the plane at (**c**) *T*=295 K, (**d**) 315 K and (**e**) 320 K, respectively. (**d**) Scale bar, 200 nm. (**f**), (**g**) and (**h**) are Fourier transforms of the LTEM images **c**, **d** and **e** respectively. (**i**) Contour plot of skyrmion density of the Co_8_Zn_9_Mn_3_ (111)-thin plate in *H* versus *T* plane as deduced from LTEM observations obtained by counting the number of skyrmions in the area of ∼120 μm^2^ at each temperature and magnetic field. Some regions within the area contained dense SkX as in image **d** but other regions did not, making the average density smaller than expected from the image **d**. In the present bulk magnetization measurement, demagnetization factor (*N*) is ∼0.1 from the sample shape, whereas *N*∼1 in the thin-plate form for LTEM observation. Taking the magnitude of the magnetization in this sample into consideration, it explains factor of 7–8 difference in the threshold external magnetic field for formation of the SkX phase in the bulk form (**b**) and thin-plate form (**i**).

The signature of the SkX formation in bulk specimens was also observed by magnetization measurements. Figure 3a shows isothermal d*M*/d*H* versus *H* curves measured at different temperatures for a polycrystalline piece of Co_8_Zn_9_Mn_3_. Between ∼311 and ∼320 K, a characteristic dip structure can be clearly seen at ∼100 Oe, signalling the formation of the SkX in analogy with the *B*20-type alloys^33^ and Cu_2_OSeO_3_ systems^21^,^22^. The magnetic phase diagram can be established from a contour plot of d*M*/d*H*, as shown in Fig. 3b. The SkX phase in the bulk appears in a relatively narrow temperature window with a width of ∼9 K just below the helical ordering temperature. These features are in accordance with the phase diagram of bulk *B*20 alloys^6^ and Cu_2_OSeO_3_ (refs 21, 22), and suggest that thermal fluctuations play a crucial role in stabilising the SkX phase also in this system of bulk form^6^.

To observe directly the SkX and uncover the influence of dimensionality on its stability, the phase diagram of a (111) thin plate (<150 nm) of Co_8_Zn_9_Mn_3_ was studied with the use of LTEM. Figures 3c–e and 3f–h show typical LTEM images and their Fourier transformed images taken at *H*=0.7 kOe. At *T*=295 K, a stripe structure with weak contrast in real space (Fig. 3c) and broad peaks in Fourier space (Fig. 3f) is observed, suggesting the system to be in the helical phase. At 315 K (Fig. 3d,g), the formation of the SkX with hexagonal symmetry is clearly observed. Further increase of the temperature to 320 K (Fig. 3e,h) results in the disappearance of magnetic contrast as the system enters the paramagnetic phase. Figure 3i shows a contour plot of the skyrmion density as a function of magnetic field versus temperature. The magnetic field range required to induce the SkX phase in the thin plate is significantly increased compared with the bulk case due to the demagnetization factor of the plate-like geometry^7^,^21^,^27^. Likewise, the SkX phase expands over a wider temperature range of ∼20 K. As was the case for the other chiral magnets, this can be interpreted in terms of a relative suppression of the competing conical phase with out-of-plane propagation vector compared with the bulk case^7^,^21^,^27^.

### Detection of the SkX formation in the bulk form

**Figure 4:**
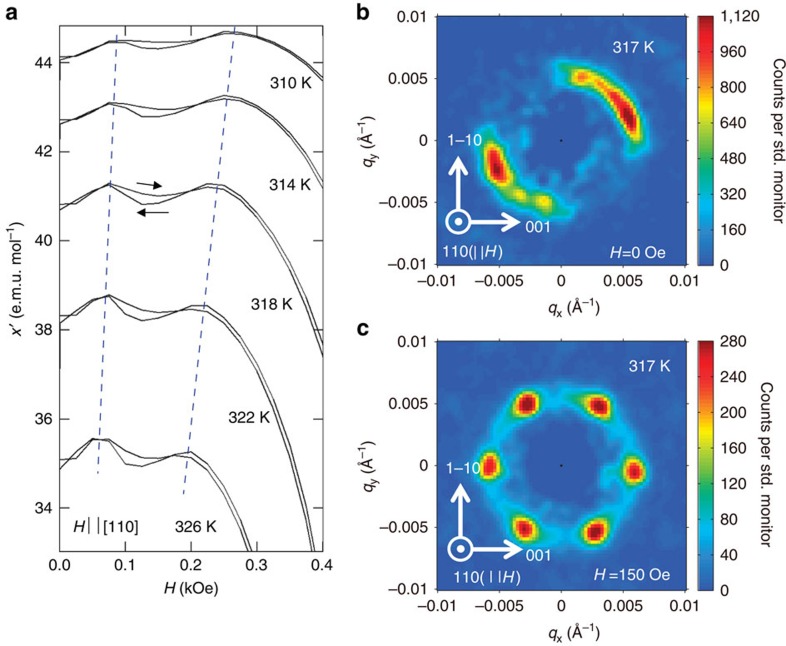
Formation of the skyrmion crystal confirmed by small-angle neutron scattering (SANS). (**a**) Temperature dependence of a.c.-magnetic susceptibility versus magnetic field measured in *H* || [110] for a single-crystalline piece of Co_8_Zn_8_Mn_4_. Hysteresis on increasing and decreasing magnetic field is indicated by arrows. The magnetic field region sandwiched by dotted lines indicates the skyrmion crystal phase. (**b**,**c**) SANS images for a single-crystalline sample of Co_8_Zn_8_Mn_4_ at 317 K obtained at *H*=0 and *H*=150 Oe, respectively. *H* and the incident neutron beam are parallel with each other and both along [110] direction. The horizontal and vertical axes of the SANS images correspond to the [001] and [1–10] crystal directions, respectively.

To confirm the formation of the SkX in the bulk form more directly, SANS measurements were performed on a single-crystalline piece of Co_8_Zn_8_Mn_4_ cut out from a polycrystalline ingot with fairly large grains obtained by the Bridgman method. Figure 4a shows the temperature dependence of the ac-susceptibility versus magnetic field along the [110] direction. We conducted SANS measurements in the configuration where the directions of incident neutron beam and magnetic field were both approximately along the [110] direction. As shown in Fig. 4b, a ring-shaped intensity distribution can be seen at *T*=317 K and *H*=0, suggesting the formation of multi-domain single *q*-helical structures with relatively flexible *q*-directions. At *H*=150 Oe, which is inside of the dip anomaly of a.c.-susceptibility (see Fig. 4a), a six-fold-symmetric SANS pattern with magnetic reflections at *q*∼0.0058 Å^−1^ can be clearly observed (Fig. 4c), thereby directly demonstrating the formation of the triple *q*-state or SkX with the lattice constant of 
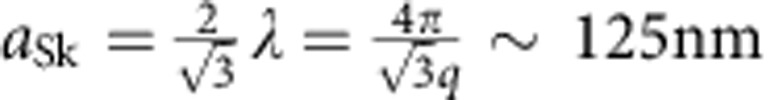
.

## Discussion

The present observation of skyrmions in this *β*-Mn-type chiral magnet demonstrates the long-held expectation that new skyrmion hosting systems can be found in a variety of non-centrosymmetric crystal symmetries, and may as such stimulate experimental exploration of other realizations, including other *β*-Mn structured materials. Likewise, the long sought-after demonstration of skyrmion stabilization above room temperature implies that complicated cooling is no longer a limiting factor for the potential integration of skyrmions into technological spintronics devices and applications.

## Methods

### Sample preparation

Polycrystalline samples of Co_*x*_Zn_*y*_Mn_*z*_ (*x*+*y*+*z*=20) were synthesized by the method described in ref. 29. Stoichiometric amounts of pure Co, Zn and Mn pieces of 2 g total mass were sealed in evacuated quartz tubes (∼10^−3^ Pa), heated to 1,000 °C for 12 h, followed by slow cooling (1 °C h^−1^) down to 925 °C, then kept at this temperature for 75–100 h. Finally, the products were water quenched. A polycrystalline sample of Co_8_Zn_8_Mn_4_ with larger grain sizes (typically up to 3 mm) was grown by the Bridgman method with cooling from 1,025 °C to ∼700 °C over 1 week, followed by water quench. A single-crystalline specimen was cut out from the resultant product and oriented with use of back-reflection X-ray Laue photographs.

### Magnetization measurements

Magnetization and a.c.-susceptibility were measured by a superconducting quantum interference device magnetometer (MPMS3, Quantum Design) equipped with oven and a.c.-susceptibility measurement options.

### SANS measurements

The long-wavelength magnetic structures in bulk samples of Co_*x*_Zn_*y*_Mn_*z*_ were measured using the SANS instrument SANS-I at the Swiss Spallation Neutron Source, Paul Scherrer Institute, Switzerland. In a typical instrument setup, neutrons of wavelength 10 Å were selected with a full-width at half-maximum spread (Δ*λ*/*λ*) of ∼10%, and collimated over a distance of 18 m before the sample. The scattered neutrons were collected by a position-sensitive multi-detector placed 18 m behind the sample. Polycrystalline ingot samples of Co_*x*_Zn_*y*_Mn_*z*_, and single crystals samples of Co_8_Zn_8_Mn_4_, were mounted inside a horizontal field cryomagnet that was installed on the beamline. The cryomagnet could be rotated so that the direction of applied magnetic field was either approximately parallel, or approximately perpendicular to the neutron beam. For SANS measurements on the polycrystalline samples, the magnetic field was applied perpendicular to the neutron beam (Fig. 1d,e), while measurements on the single crystal samples made use of an applied field approximately parallel to the neutron beam (Fig. 4b,c).

For either experimental geometry, the SANS measurements were done typically by rotating the sample and cryomagnet ensemble in a step-wise manner around its vertical axis—a so-called rocking scan—and over a sufficiently broad angular range, so as to move the various magnetic diffraction spots through the Bragg condition at the detector. Typical rocking scans spanned an angular range of up to ±20° with a SANS measurement conducted every 2°. This chosen step size was larger than the calculated instrumental contribution to the rocking half-width of the magnetic Bragg peaks (∼0.2°), yet smaller than the observed Lorentz factor-corrected rocking half-widths, which, while sample dependent, were always >∼4.5°. This observation demonstrates the observed rocking widths of the magnetic peaks to be dominated by the mosaicity of the magnetic order in the sample. By summing the detector measurements taken over all rotation angles, all of the diffraction spots can be seen in a single image, thus resulting in the SANS diffraction patterns shown in Figs 1 and 4.

### Determination of the crystallographic chirality

The crystal chirality of Co_8_Zn_10_Mn_2_ was determined by using CBED pattern. The CBED pattern was obtained by using a JEM-2100F at an accelerating voltage of 200 kV at room temperature. For the simulation of CBED patterns, the MBFIT software^34^ was used. From the comparison between the experimental and simulated CBED patterns, the crystal chirality can be determined uniquely.

### LTEM measurements

For LTEM measurements, thin plates were cut from polycrystalline samples and thinned by mechanical polishing and argon ion milling. The crystal orientation and thickness were confirmed by conventional TEM observation and the electron energy-loss spectroscopy, respectively. In LTEM observations, the magnetic structures can be imaged as convergences (bright contrast) or divergences (dark contrast) of the electron beam on the defocused (under- or over focused) image planes. The inversion of contrast between the over- and under-focused images as well as disappearance of the contrast above *T*_c_ assured the magnetic origin of the image contrast. The analysis of the in-plane components of magnetic induction (which reflects the in-plane magnetization) from the LTEM data was performed by the transport-of-intensity equation method with use of the QPt software package (HREM Co.)^32^.

### Drawing of the crystal structure

The crystal structures in Fig. 1a,b were drawn with the use of VESTA^35^.

## Additional information

**How to cite this article:** Tokunaga, Y. *et al.* A new class of chiral materials hosting magnetic skyrmions beyond room temperature. *Nat. Commun.* **6**:7638 doi: 10.1038/ncomms8638 (2015).

## References

[b1] Skyrme T. Unified field theory of mesons and baryons. Nucl. Phys. 31, 556–569 (1962).

[b2] Bogdanov A. N. & Yablonskii D. A. Thermodynamically stable ‘vortices' in magnetically ordered crystals. The mixed state of magnets. Sov. Phys. JETP 68, 101–103 (1989).

[b3] Nagaosa N. & Tokura Y. Topological properties and dynamics of magnetic skyrmions. Nat. Nanotechol. 8, 899–911 (2013). 10.1038/nnano.2013.243 24302027

[b4] Lin Y. S., Grundy J. & Giess E. A. Bubble domains in magnetostatically coupled garnet films. Appl. Phys. Lett. 23, 485–487 (1973).

[b5] Yu X. Z. *et al.* Magnetic stripes and skyrmions with helicity reversals. Proc. Natl Acad. Sci. USA 109, 8856–8860 (2012). 22615354 10.1073/pnas.1118496109 PMC3384203

[b6] Mühlbauer S. *et al.* Skyrmion lattice in a chiral magnet. Science 323, 915–919 (2009). 19213914 10.1126/science.1166767

[b7] Yu X. Z. *et al.* Real-space observation of a two-dimensional skyrmion crystal. Nature 465, 901–904 (2010). 20559382 10.1038/nature09124

[b8] Dzyaloshinskii I. A thermodynamic theory of weak ferromagnetism of antiferromagnetics. J. Phys. Chem. Solids 4, 241–255 (1958).

[b9] Moriya T. Anisotropic superexchange interaction and weak ferromagnetism. Phys. Rev. 120, 91–98 (1960).

[b10] Okubo T., Chung S. & Kawamura H. Multiple-*q* states and the skyrmion lattice of the triangular-lattice Heisenberg antiferromagnet under magnetic fields. Phys. Rev. Lett. 108, 017206 (2012). 22304286 10.1103/PhysRevLett.108.017206

[b11] Heinze S. *et al.* Spontaneous atomic-scale magnetic skyrmion lattice in two dimensions. Nat. Phys. 7, 713–718 (2011).

[b12] Lee M., Kang W., Onose Y., Tokura Y. & Ong N. P. Unusual Hall anomaly in MnSi under pressure. Phys. Rev. Lett. 102, 186601 (2009). 19518894 10.1103/PhysRevLett.102.186601

[b13] Neubauer A. *et al.* Topological Hall effect in the A phase of MnSi. Phys. Rev. Lett. 102, 186602 (2009). 19518895 10.1103/PhysRevLett.102.186602

[b14] Zhang J., Mostovoy M., Han J. H. & Nagaosa N. Dynamics of skyrmion crystals in metallic thin films. Phys. Rev. Lett. 107, 136804 (2011). 22026888 10.1103/PhysRevLett.107.136804

[b15] Jonietz F. *et al.* Spin transfer torques in MnSi at ultralow current densities. Science 330, 1648–1651 (2010). 21164010 10.1126/science.1195709

[b16] Schulz T. *et al.* Emergent electrodynamics of skyrmions in a chiral magnet. Nat. Phys. 8, 301–304 (2012).

[b17] Yu X. Z. *et al.* Skyrmion flow near room temperature in ultra-low current density. Nat. Commun. 3, 988 (2012). 22871807 10.1038/ncomms1990

[b18] Everschor K. *et al.* Rotating skyrmion lattices by spin torques and field or temperature gradients. Phys. Rev. B 86, 054432 (2012).

[b19] Iwasaki J., Mochizuki M. & Nagaosa N. Universal current–velocity relation of skyrmion motion in chiral magnets. Nat. Commun. 4, 1463 (2013). 23403564 10.1038/ncomms2442

[b20] Iwasaki J., Mochizuki M. & Nagaosa N. Current-induced skyrmion dynamics in constricted geometries. Nat. Nanotechol. 8, 742–747 (2013). 10.1038/nnano.2013.176 24013132

[b21] Seki S. *et al.* Observation of skyrmions in a multiferroic material. Science 336, 198–201 (2012). 22499941 10.1126/science.1214143

[b22] Adams T. *et al.* Long-wavelength helimagnetic order and skyrmion lattice phase in Cu_2_OSeO_3_. Phys. Rev. Lett. 108, 237204 (2012). 23003986 10.1103/PhysRevLett.108.237204

[b23] Seki S., Ishiwata S. & Tokura Y. Magnetoelectric nature of skyrmions in a chiral magnetic insulator Cu_2_OSeO_3_. Phys. Rev. B 86, 060403 (2012).

[b24] White J. S. *et al.* Electric-Field-Induced Skyrmion Distortion and Giant Lattice Rotation in the Magnetoelectric Insulator Cu_2_OSeO_3_. Phys. Rev. Lett. 113, 107203 (2014). 25238382 10.1103/PhysRevLett.113.107203

[b25] Omrani A. A. *et al.* Exploration of the helimagnetic and skyrmion lattice phase diagram in Cu_2_OSeO_3_ using magnetoelectric susceptibility. Phys. Rev. B 89, 054406 (2014).

[b26] Bogdanov A. N., Rößler U. K., Wolf M. & Müller K.-H. Magnetic structures and reorientation transitions in noncentrosymmetric uniaxial antiferromagnets. Phys. Rev. B 66, 214410 (2002).

[b27] Yu X. Z. *et al.* Near room-temperature formation of a skyrmion crystal in thin-films of the helimagnet FeGe. Nat. Mater. 10, 106–109 (2011). 21131963 10.1038/nmat2916

[b28] Buschow K. H. J., van Engen P. G. & Jongebreur R. Magneto-optical properties of metallic ferromagnetic materials. J. Magn. Magn. Mater. 38, 1–22 (1983).

[b29] Xie W. *et al.* *β*-Mn-type Co_8+*x*_Zn_12–*x*_ as a defect cubic laves phase: site preferences, magnetism, and electronic structure. Inorg. Chem. 52, 9399–9408 (2013). 23909791 10.1021/ic4009653

[b30] Hori T., Shiraish H. & Ishii Y. Magnetic properties of *β*-MnCoZn alloys. J. Magn. Magn. Mater. 310, 1820–1822 (2007).

[b31] Yu X. Z. *et al.* Variation of skyrmion forms and their stability in MnSi thin plates. Phys. Rev. B 91, 054411 (2015).

[b32] Ishizuka K. & Allman B. Phase measurement of atomic resolution image using transport of intensity equation. J. Electron Microsc. 54, 191 (2005). 10.1093/jmicro/dfi024 16076863

[b33] Thessieu C., Pfleiderer C., Stepanov A. N. & Flouquet J. Field dependence of the magnetic quantum phase transition in MnSi. J. Phys. Cond. Matter. 9, 6677–6688 (1997).

[b34] Tsuda K. & Tanaka M. Refinement of crystal structural parameters using two-dimensional energy-filtered CBED patterns. Acta Cryst. A55, 939–954 (1999). 10.1107/s0108767399005401 10927304

[b35] Momma K. & Izumi F. VESTA 3 for three-dimensional visualization of crystal, volumetric and morphology data. J. Appl. Cryst. 44, 1272–1276 (2011).

